# Novel Hyaluronate Lyase Involved in Pathogenicity of *Streptococcus dysgalactiae* subsp. *equisimilis*

**DOI:** 10.3389/fmicb.2020.552418

**Published:** 2020-09-24

**Authors:** Van An Nguyen, Kohei Ogura, Miki Matsue, Norihiko Takemoto, Kanae Mukai, Yukari Nakajima, Thuy Linh Hoang, Yasunori Iwata, Norihiko Sakai, Takashi Wada, Wataru Hashimoto, Shigefumi Okamoto, Hiroshi Ichimura

**Affiliations:** ^1^ Department of Viral Infection and International Health, Graduate School of Medical Sciences, Kanazawa University, Kanazawa, Japan; ^2^ Advanced Health Care Science Research Unit, Institute for Frontier Science Initiative, Kanazawa University, Kanazawa, Japan; ^3^ Department of Clinical Laboratory Science, Faculty of Health Sciences, Institute of Medical, Pharmaceutical and Health Sciences, Kanazawa University, Kanazawa, Japan; ^4^ Pathogenic Microbe Laboratory, Research Institute, National Center for Global Health and Medicine, Shinjuku, Japan; ^5^ Department of Nursing Sciences, Faculty of Health Sciences, Institute of Medical, Pharmaceutical and Health Sciences, Kanazawa University, Kanazawa, Japan; ^6^ Department of Nephrology and Laboratory Medicine, Kanazawa University, Kanazawa, Japan; ^7^ Division of Infection Control, Kanazawa University, Kanazawa, Japan; ^8^ Department of Clinical Laboratory, Kanazawa University Hospital, Kanazawa, Japan; ^9^ Laboratory of Basic and Applied Molecular Biotechnology, Division of Food Science and Biotechnology, Graduate School of Agriculture, Kyoto University, Uji, Japan

**Keywords:** *Streptococcus dysgalactiae* subsp. *equisimilis*, hyaluronate, hyaluronate lyase, pathogenicity, *Streptococcus agalaciate*, skin pH

## Abstract

*Streptococcus dysgalactiae* subsp. *equisimilis* (SDSE) causes cellulitis, bacteremia, and invasive diseases, such as streptococcal toxic shock syndrome. Although SDSE infection is more prevalent among elderly individuals and those with diabetes mellitus than infections with *Streptococcus pyogenes* (Group A streptococci; GAS) and *Streptococcus agalactiae* (Group B streptococci; GBS), the mechanisms underlying the pathogenicity of SDSE remain unknown. SDSE possesses a gene *hylD* encoding a hyaluronate lyase (HylD), whose homologue (HylB) is involved in pathogenicity of GBS, while the role of HylD has not been characterized. In this study, we focused on the enzyme HylD produced by SDSE; HylD cleaves hyaluronate (HA) and generates unsaturated disaccharides *via* a β-elimination reaction. Hyaluronate-agar plate assays revealed that SDSE promoted dramatic HA degradation. SDSE expresses both HylD and an unsaturated glucuronyl hydrolase (UGL) that catalyzes the degradation of HA-derived oligosaccharides; as such, SDSE was more effective at HA degradation than other β-hemolytic streptococci, including GAS and GBS. Although HylD shows some homology to HylB, a similar enzyme produced by GBS, HylD exhibited significantly higher enzymatic activity than HylB at pH 6.0, conditions that are detected in the skin of both elderly individuals and those with diabetes mellitus. We also detected upregulation of transcripts from *hylD* and *ugl* genes from SDSE wild-type collected from the mouse peritoneal cavity; upregulated expression of *ugl* was not observed in *ΔhylD* SDSE mutants. These results suggested that disaccharides produced by the actions of HylD are capable of triggering downstream pathways that catalyze their destruction. Furthermore, we determined that infection with SDSE*ΔhylD* was significantly less lethal than infection with the parent strain. When mouse skin wounds were infected for 2 days, intensive infiltration of neutrophils was observed around the wound areas infected with SDSE wild-type but not SDSE*ΔhylD*. Our investigation suggested that HylD and UGL play important roles in nutrient acquisition from hosts, followed by the bacterial pathogenicity damaging host tissues.

## Introduction

*Streptococcus pyogenes* (Group A streptococci; GAS), *Streptococcus agalactiae* (Group B streptococci; GBS), and *Streptococcus dysgalactiae* subsp. *equisimilis* (SDSE) are β-hemolytic streptococci. Takahashi et al. (2011) reported that SDSE had Lancefield group G or C antigens on their cell surface, whereas our group recently reported on the prevalence of Lancefield group A SDSE in Japan (Ishihara et al., 2020). SDSE colonizes the pharynx, gastrointestinal tract, female genital tract, and skin as a commensal microorganism (Yung et al., 2019) and is involved in a wide variety of infectious diseases from relatively minor wound infections, erysipelas, and cellulitis to life-threatening necrotizing fasciitis and streptococcal toxic shock syndrome (STSS; Brandt and Spellerberg, 2009; Rantala, 2014). Genomic analysis revealed that several virulence factors are conserved in both GAS and SDSE (Shimomura et al., 2011). However, SDSE lacks several virulence factors, including the streptococcal pyrogenic exotoxin (*spe*) and hyaluronate (HA) synthesis (*hasA* and *hasB*) genes. GAS and GBS produce and secrete HA that forms a bacterial capsule that plays a critical role in promoting immune evasion and pathogenicity (Stollerman and Dale, 2008). Transcription of the GAS HA biosynthesis operon is regulated by a two-component CovR-CovS (also known as CsrR-CsrS) regulatory system; loss of CovR-CovS function results in overexpression of HA (Heath et al., 1999; Falaleeva et al., 2014). SDSE does not produce HA, and as such, deletion of this regulatory system from these bacteria does not result in the generation of mucoid colonies (Watanabe et al., 2013). Of interest, we recently reported that SDSE utilizes cell wall-anchoring proteins as envelopes in serum-containing medium (Matsue et al., 2020).

**Figure 1:**
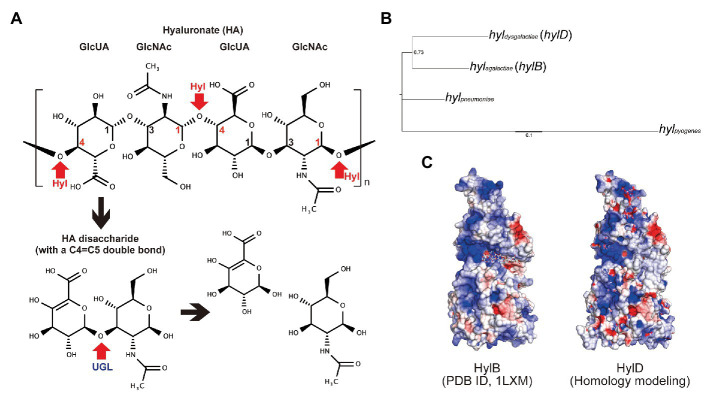
Streptococcal hyaluronate lyases. **(A)** Schematic diagram of hyaluronate (HA) degradation of Hyl and UGL. β1,4 linkages between glucuronic acid (GlcUA) and N-acetylglucosamine (GlcNAc) were degraded by hyaluronate lyase (Hyl; represented in red arrows). The resultant unsaturated HA disaccharides were hydrolyzed by unsaturated glucuronyl hydrolase (UGL; represented by a red arrow). The chemical formula was drawn by MarvinSketch software (ChemAxon). **(B)** Genetic diversity of streptococcal hyaluronate lyases (Hyls) from *S. pyogenes* strain 476 (locus tag: M1GAS476_0819), *Streptococcus pneumoniae* strain R6CIB17 (E5Q10_01570), *Streptococcus agalactiae* strain COH1 (GBSCOH1_1103), and *Streptococcus dysgalactiae* subsp. *equisimilis* (SDSE) strain 124 (SDEG_0654). **(C)** Structural similarity of HylB (PDB ID, 1LXM) and HylD (homology modeling using 1LXM) from *S. agalactiae* and SDSE, respectively.

In the human tissues, HA is a critical component of the extracellular matrix and functions by retaining water molecules (Baumann, 2007). Approximately 50% of human HA is associated with the integumentary system (Reed et al., 1988; Juhlin, 1997) where it functions to create a semi-permeable barrier. Some of streptococcal strains are capable of degrading HA *via* the production of hyaluronate lyases (Hyls; ENZYME entry: EC 4.2.2.1); Hyls are enzymes that cleave β1,4 linkages in HA *via* a β-elimination reaction (Hynes, 2004; Figure 1A). Unsaturated HA oligosaccharides produced by the actions of Hyls are further degraded by unsaturated glucuronyl hydrolase (UGL), an enzyme that catalyzes the hydrolysis of oligosaccharides with unsaturated glucuronyl residues at their non-reducing termini (Hashimoto et al., 1999; Maruyama et al., 2009). *hylP* genes encoding active Hyls in GAS strains were detected in specific bacteriophages, whereas clinical isolates of SDSE encode *hylD* genes that are homologous to *hylB* genes from *S. agalactiae* (Shimomura et al., 2011). Hyaluronate lyase from GBS (HylB) functions as a virulence factor in the human tissues. Interestingly, HylB-derived HA disaccharides resulted in reduced levels of pro-inflammatory cytokine when administered as treatment in a mouse acute lung injury model; these results suggest that GBS can degrade host-generated pro-inflammatory HA fragments *via* the actions of HylB and thereby may escape from host immune-mediated detection (Kolar et al., 2015).

*Streptococcus dysgalactiae* subsp. *equisimilis* has been identified as the etiologic agent in severe invasive streptococcal infections in the elderly (Wajima et al., 2016); it is unclear what promotes this apparent high pathogenicity compared with that seen in infections with GAS and GBS. GAS and GBS both produce and degrade HA, whereas SDSE is capable of HA degradation only; these results suggest that SDSE-mediated regulation of *hyl* and its downstream factors proceed differently from that identified in GAS and GBS. Furthermore, aging changes the characteristics of the skin; among the best characteristics of these changes are the reduced production of sebum and higher pH (Choi et al., 2007; Levin and Maibach, 2008). The skin pH of patients with diabetes mellitus is also higher than that detected in healthy subjects (Behm et al., 2012). Interestingly, Meyrer et al. reported that HA concentration and polymer size of HA in the human skin do not undergo change as a function of age (Meyer and Stern, 1994). On the basis of these characteristics, we hypothesized that the pathogenicity of SDSE might relate to its capacity to degrade HA. In this study, we focused on the role of HylD, the hyaluronate lyase from SDSE, and explored its role in promoting pathogenicity. Toward this end, we examined the expression of *hylD* in bacteria grown in culture medium and in the intraperitoneal cavity of the infected mice. We measured the pH-dependent enzymatic activity of HylD and HylB and examined the impact of *hylD* gene deletion on SDSE-mediated lethality in infected mice.

## Materials and Methods

### Phylogenetic Analysis

Phylogenetic analysis was conducted using MEGA 5 software with neighbor joining method (Tamura et al., 2011) for streptococcal Hyls from *S. pyogenes* strain 476 (locus tag: M1GAS476_0819), *Streptococcus pneumoniae* strain R6CIB17 (E5Q10_01570), *S. agalactiae* strain COH1 (GBSCOH1_1103), and SDSE strain 124 (SDEG_0654). Tree was visualized by Fig Tree.^1^

### Homology Modeling

The protein structure of HylD was predicted by UCSF Chimera and MODELER using HylB (PDB ID, 1LXM) as a template (Pettersen et al., 2004; Eswar et al., 2006). Protein surface and electric charge were visualized by CueMol software (Molecular Visualization Framework; http://www.cuemol.org/). Isoelectric points (pIs) were computed based on their primary protein structure (amino acid sequences) using the ExPASy website (Bjellqvist et al., 1993).

### Bacterial Strains

Bacterial strains SDSE-124 (GenBank accession number AP010935.1), SDSE-C167 (AP012976.1), GAS M1 476 (AP012491.2), GBS COH1 (HG939456.1), and GBS A909 (CP000114.1) were utilized in this study. Additional bacterial strains included TPCH-F36, which was isolated in Toyama Prefecture Central Hospital (Japan); KNZ01, KNZ03, KNZ10, and KNZ16 from Kanazawa University Hospital, Japan (Matsue et al., 2020); and GAS M1 SMD from National Center of Global Health and Medicine (Japan). The bacteria utilized in this study were cultured in Todd Hewitt broth (Becton Dickinson, Franklin Lakes, NJ, USA) supplemented with 0.2% yeast extract (THY, Becton Dickinson) or brain-heart infusion (BHI, Becton Dickinson); bacterial cultures were grown overnight at 37°C in a 5% CO_2_ atmosphere. All experiments were conducted in accordance with the WHO Laboratory Biosafety Manual and institutional safety procedures at Kanazawa University.

### Preparation of the ΔhylD Mutant Strain

*hylD* gene in SDSE-124 (locus tag: SDEG_0654) was deleted using thermosensitive suicide vector pSET4S according to a previous study (Takamatsu et al., 2001; Supplementary Figure S1). One kilo byte upstream (620,723-621,724) and 1 kb downstream (624,929-625,928) sequence of the *hylD* gene were concatenated by overlap extension PCR using Gflex DNA polymerase (Takara Bio, Japan) and cloned into EcoRI-digested pSET4S using an In-Fusion HD Cloning Kit (Takara Bio). SDSE-124 was transformed by electroporation with the constructed plasmid. After incubation at 28°C for 4 h, the cells were spread on a 250-μg/ml spectinomycin-containing BHI (Sp250-BHI) plate, incubated at 28°C for 2 days, and collected in Sp250-BHI medium. Single-crossover spectinomycin-resistant mutant cells were obtained after incubation in Sp250-BHI medium and spreading on Sp250-BHI plates at 28°C. The single-crossover mutants were repeatedly incubated at 28°C in BHI (no spectinomycin). The spectinomycin-sensitive colonies were considered to be double-crossover strains with Δ*hylD*. The single- and double-crossovers were confirmed by colony direct PCR using Gflex DNA polymerase. The resultant sequences were confirmed using an Applied Biosystems 3130xl Genetic Analyzer.

### Hyaluronate Degradation Halo Assay

Degraded HA halo assays were performed as previously described with some modifications (Smith and Willett, 1968; Oiki et al., 2019). Agar plates consisting of 0.5% peptone (Becton Dickinson, Franklin Lakes, NJ, USA), 0.3% beef extract (Becton Dickinson, Franklin Lakes, NJ, USA), 1% agar, 1% bovine serum albumin (Nacalai Tesque, Japan), and 0.4 mg/ml hyaluronic acid (FUJIFILM Wako Pure Chemical Corporation, Japan) were prepared. Bacterial strains were grown in BHI medium, washed with fresh BHI, and resuspended in fresh BHI at the indicated optical density at 600 nm (OD_600_). One microliter of bacterial suspensions at the indicated concentration was spotted on the peptone agar, followed by overnight incubation at 37°C in a 5% CO_2_ atmosphere. The plates were then flooded with 2 N acetic acid and allowed to stand for 10 min. Clear regions demarcate zones of HA degradation.

### Quantitative Real-Time PCR

Bacteria were collected in RNAprotect Bacteria Reagent (Qiagen, Hilden, Germany). After treatment with 20 mg/ml lysozyme (FUJIFILM Wako Pure Chemical Corporation) and 50 U/ml mutanolysin (Merck) at 37°C for 90 min, bacterial RNAs were extracted with NucleoSpin RNA kit (Macherey-Nagel, Düren, Germany), treated with gDNA Eraser (Takara Bio, Japan), and reverse-transcribed with PrimeScript RT reagent Kit according to the manufacturers’ protocols. Quantitative real-time PCR (RT-qPCR) was conducted in AriaMx Real-Time PCR System (Agilent Technologies) using a QuantiFast SYBR Green PCR Kit (Qiagen) or a QuantiTect Probe PCR Kit (Qiagen). Primers and probes for quantification of *hyl*, *ugl*, and *mutS* expression are listed in Table 1. The oligonucleotides were purchased from Eurofin Genomics, Japan. Standard curves for quantitative analysis of *hyl*, *ugl*, and *mutS* gene expression were prepared with the indicated copy numbers of genes inserted into plasmids pQE30/hylB, pQE30/hylD, pQE30/GBS_ugl, and pQE30/SDSE_ugl.

**Table 1:** Primers and probes used in this study.

Bacteria/Genes	Forward and reverse primer sequences (5′ > 3′)	Probe
For SYBR Green qPCR (Figure 2C)		
SDSE-124/*hyl*	CCATTGATCAGCGAAAGGAT, AGACCAGCGTCCTGACTGTT	
SDSE-124/*ugl*	AATCCTTAATCAAGGCTCTTAGCAT, GGATTTAATTTTTGGAGATGGAAGT	
GBS-COH1/*hyl*	GAGTCTGGCTTGGCTTCATC, CACCAAGGGCTTTAAATGGA	
GBS-COH1/*ugl*	GTGTCACACGACAGGGTTATAGTG, ATGTATCTCTAGGCTGACCCGA	
SDSE-124&GBS-COH1/*mutS*	CCAGATGCTTTTTTGCTTTTTAG, ACCTCACGCTTGACCAC	
For Probe qPCR (Figure 5A)		
SDSE-124/*hyl*	CGACTTCAACAGACAAAG, CAGGATTAATGACAACCG	FAM-AACTTCTTGCCTGCTTCATTGGTC-BQH1
SDSE-124/*ugl*	GGCATTCCTTTGACCTATC, TCGTGGATACCACATACG	FAM-AACTATCACGAGACTGTCCACTTCC-BQH1
SDSE-124/*mutS*	CTTGCAATACGTTCACAA, CTAGCATTCTCTACCAAATC	FAM-AATGCGAGAACTCAGCCACTTG-BQH1

### Growth Assays

The chemically defined medium (CDM) was prepared as previously described (van de Rijn and Kessler, 1980) and supplemented with HA (0.4 mg/ml), HA (2.0 mg/ml), or glucose (10 mg/ml). The amounts of carbon sources were chosen according to HA concentration in human skin (normally approximately 0.4 mg/ml), and knee joint HA (2.0 mg/ml) and fasting blood glucose (10 mg/ml) concentrations (Balazs et al., 1967; Dahl et al., 1985; Tammi et al., 1994). All media were filtered through a 0.45 μm-pore-size filter prior to use. SDSE-124, SDSE-124 *ΔhylD*, GBS-COH1, and GAS-476 were grown in THY medium, washed with fresh CDM with no added carbohydrate [CDM (−)], and resuspended in fresh CDM (−). A specific amount of bacterial suspension was transferred into 3 ml of CDM with or without added carbohydrate to achieve an optical density of 0.05 at 600 nm. These bacterial suspensions were incubated at 37°C in a 5% CO_2_ atmosphere. OD_600_ was measured with a Genesys 20 spectrophotometer (Thermo Scientific, USA) and the bacterial growth in each medium was assessed in three independent tests, in triplicate.

### Overexpression of Hyl and UGL

Hyaluronate lyases from GBS-COH1 and SDSE-124 were overexpressed in *Escherichia coli*. We could not obtain full-length Hyl. Since a previous report showed that Hyl undergoes an autocatalytic conversion to a smaller enzymatically active form (Jedrzejas and Chantalat, 2000), we utilized truncated versions of HylB and HylD, both with N-terminal 170 amino acid deletions. The *hyl* and *ugl* genes were cloned into pCold I (Takara Bio) using a Gibson Assembly Kit (New England Biolabs, USA). BL21(DE3) was transformed with the resultant plasmid, cultivated, and homogenized for recombinant protein purification with TALON Metal Affinity Resin (Takara Bio). After buffer exchange with 5 mM HEPES-NaOH (pH 7.0) using PD-10 columns (GE Healthcare, USA), the purified recombinant protein solutions were utilized for enzymatic assays. Protein concentrations were measured by BCA Protein Assay kit (ThermoFisher Scientific).

### Enzymatic Activity of HylB and HylD

Ten microliters of the enzyme (recombinant HylB or HylD) solution (0.05 mg/ml) were mixed with 500 μl of 1 mg/ml HA solution containing 0.2 M buffer (described below) and left at 37°C. The increase in absorbance at 235 nm due to C=C double bond formation was monitored with a U-3210 spectrometer (Hitachi Industries, Japan) at a final pH of 5.0 (in the presence of citrate buffer), 5.5 (citrate), 6.0 (MOPS-NaOH), 6.5 (MOPS-NaOH), 7.0 (MOPS-NaOH), 7.5 (HEPES-NaOH), 8.0 (HEPES-NaOH), 8.5 (glycine-NaOH), or 9.0 (glycine-NaOH).

### Product Analysis With Thin Layer Chromatography

Five microliters of recombinant HylD and/or UGL solution (0.1 mg/ml) was mixed with 100 μl of 1 mg/ml HA solution containing 0.1 M citrate buffer pH 6.0 and left overnight at 37°C. Ten microliters of the solutions were spotted on a silicagel 70 TLC plate (FUJIFILM Wako Pure Chemical Corporation). The resulting products were separated with thin layer chromatography using a 1-butanol/acetic acid/water (4:1:1, v/v) solvent system. The products were visualized after spraying them with 5% sodium phosphomolybdate n-hydrate (FUJIFILM Wako Pure Chemical Corporation) in ethanol and heating to 200°C for 5 min.

### Mouse Intraperitoneal Injection Assays

*Streptococcus dysgalactiae* subsp. *equisimilis* strains were cultured in BHI. Bacterial cells were harvested by centrifugation and washed with ice-cold fresh BHI when the culture was in its exponential phase (OD_600_ = 0.5). A 0.5-ml aliquot containing approximately 2.0 × 10^6^ colony-forming units (CFU) of SDSE-124 (WT or Δ*hylD*) was used for intraperitoneal injection and the bacteria were recovered *via* injection of 5 ml of fresh BHI at the indicated time points. To determine survival rates, 5 week-old C57BL/6 J mice (Sankyo Laboratory Service Corp., Inc., Tokyo, Japan) were injected intraperitoneally with 0.5 ml containing approximately 2.0 × 10^6^ CFU of SDSE strains and observed once a day for 7 days according to a previous study (Watanabe et al., 2013). The volume for administration was determined according to a guideline (Diehl et al., 2001).

### Murine Wound Model

Skin wounds were made according to a previous report with some modifications (Mukai et al., 2016), 5 week-old C57BL/6JJ mice were anesthetized through intraperitoneal administration of a mixture of Dormicum (4 mg/kg; Astellas Pharma, Inc., Tokyo, Japan), Vetorphale (5 mg/kg; Meiji Seika Pharma Co., Ltd., Tokyo, Japan), and Domitor (0.3 mg/kg; Nippon Zenyaku Kogyo Co., Ltd., Fukushima, Japan). The backs of the mice were shaved, and their skin was disinfected with ethanol. A circular full-thickness wound (4 mm in diameter) including the panniculus carnosus muscle was made using a sterile biopsy punch (Kai Industries Co. Ltd., Gifu, Japan). The wounds were covered with a transparent dressing (Tegaderm; 3 M Health Care, Tokyo, Japan) for 24 h to maintain a moist environment. Then, a 0.1-ml aliquot containing approximately 1.4 × 10^6^ CFUs of SDSE-124 (WT or Δ*hylD*) in PBS was inoculated directly onto the wounds of the mice under anesthesia, which were then covered with sterile gauze and transparent dressing. On day 2 post-infection, the infected mice were anesthetized with Sevofrane (Maruishi Pharmaceutical Co., Ltd., Osaka, Japan); then, blood was immediately collected for determination of Interleukin 6 levels (R&D Systems, Minneapolis, MN, USA), and tissue was excised from the skin lesion, fixed in 4% paraformaldehyde in PBS, and then embedded in paraffin. The tissue samples were stained with hematoxylin and eosin (H&E; Muto Pure Chemicals Co., Ltd., Tokyo, Japan) and Gram stain (Nissui Pharma Medical Sales Co., Ltd., Japan) according to the manufacturer’s instructions. An optical microscope (Eclipse E600 with U-III Film Camera System; Nikon Corp., Tokyo, Japan) was used to examine the stained tissue sections and images were captured using the NIS-Elements imaging software (Nikon).

### Ethics Statement

All animal experiments were conducted in accordance with the Declaration of Helsinki and Act on Welfare and Management of Animal (Ministry of Environment, Japan). All genetic recombination experiments were approved by the Committee for Genetic Recombination Experiments (Kanazawa University).

## Results

### Genetic and Structural Characteristics of SDSE Hyaluronate Lyase

As shown in the phylogenetic tree (Figure 1B), SDSE hyaluronate lyase gene (*hylD*, *hyldysgalactiae*) is closely related to the *hylB* gene of GBS *hyl* (*hylB*, *hylagalactiae*) and more distant from those encoded in the genomes of GAS (*hylpyogenes*) or *S. pneumoniae hyl* (*hylpneumoniae*). Since the protein structure of SDSE Hyl (HylD) has not been determined, we generated a homology modeling prediction on the basis of the known structure of GBS Hyl (HylB), which has a 44% amino acid sequence identity to HylD. Although the computed pI of HylD was 5.4 and thus substantially different from that calculated for HylB (pI = 8.8), the predicted charges on the HylD protein surface were similar to those shown for HylB (Figure 1C). Interestingly, there were significant differences in the loop region (_804_KKLTID_809_), which includes the HA binding cleft (Supplementary Figure S2).

### Degradation of Hyaluronate by SDSE

**Figure 2:**
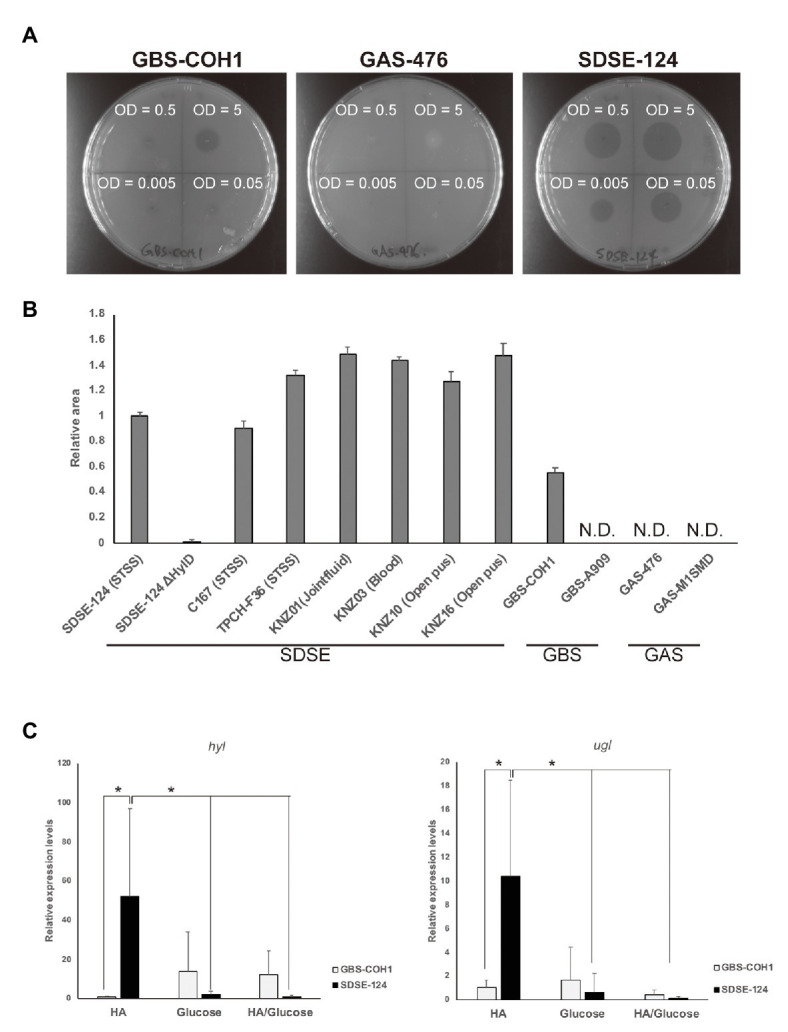
Hyaluronate degradation. **(A)** Hyaluronate degradation on agar plates. GAS-476, GBS-COH1, and SDSE-124 were grown on brain-heart infusion (BHI) medium overnight and resuspended in fresh BHI at the indicated OD_600_. One microliter of each bacterial solution was spotted on hyaluronate-containing agar plates. By soaking the plates with 2 N acetic acid, degraded hyaluronate was detected around the bacterial spots after overnight culture. **(B)** Relative area after spotting 1 μl of OD_600_ = 0.5 solution followed by overnight culture. The areas were measured using Image J software. **(C)** Quantitative evaluation of *hyl* and *ugl* gene expression after 6 h of incubation in 0.4 mg/ml hyaluronate (HA)- and/or 10 mg/ml glucose-containing medium without agar. The average and standard deviation were calculated from three separate experiments performed in quadruplicate. Data were normalized to expression levels of the *mutS* gene. Relative expression levels were calculated as 1 using the averages of the *hyl* and *ugl* expression of GBS-COH1 in HA-containing medium. Values of *p* were calculated using Student’s *t*-test; ^*^ *p* < 0.01.

We first analyzed HA degradation by GAS-476, GBS-COH1, and SDSE-124 on hyaluronate-containing agar plates. As shown in Figure 2A, SDSE-124 exhibited high levels of HA degradation at comparatively low concentrations (OD_600_ = 0.05 and 0.005). We detected degraded HA on GBS-COH1 plates only at the high concentrations (OD_600_ = 5 and 0.5) and only partially degraded HA at the highest concentration of GAS-476 (OD_600_ = 5). To determine whether the capacity to degrade HA varied depending on the specific isolate, several SDSE, GBS, and GAS strains were spotted at OD_600_ = 0.5 (Figure 2B). Significant HA degradation was observed on plates spotted with SDSE strains from patients with streptococcal toxic shock syndrome (strains C167 and TPCH-F36), as well as those isolated from joint fluid (strain KNZ01), blood (strain KNZ03), and open wounds (strains KNZ10 and KNZ16); these results indicated that all SDSE strains could degrade HA. By contrast, deletion of *hylD* gene (Δ*hylD*) in SDSE-124 completely eliminated the capacity for HA degradation. Interestingly, although SDSE TPCH-F36 expresses a phage-related hyaluronate lyase gene encoding HylP (WP_003058192.1), its capacity to degrade HA was not significantly higher than that of the KNZ01, KNZ03, KNZ10, or KNZ16 strains; these results suggested that the expression or activity of HylD is more significant than that of HylP in this SDSE strain. Although GBS-COH1 could degrade HA at high concentrations, the area of HA degradation surrounding the GBS-COH1 spots was significantly smaller compared with those containing the SDSE strains. No HA degradation was observed surrounding the spots containing GBS-A909, GAS-476, or GAS-M1SMD at OD_600_ = 0.5.

We then proceeded to compare expression levels of the genes encoding *hyl* and *ugl* (UGL, ENZYME entry EC 3.2.1.179). SDSE-124 and GBS-COH1 bacteria were cultured in hyaluronate- and/or glucose-containing medium without agar and collected for RT-qPCR analysis. As shown in Figure 2C, expression of both *hyl* and *ugl* was detected at dramatically higher levels in SDSE-124 compared with GBS-COH1. These results suggest that SDSE can express comparatively large amounts of both Hyl and UGL that facilitate rapid HA degradation. The high expression of *hyl* and *ugl* in SDE-124 was not observed in glucose-containing medium, indicating that HA acts as a signal to produce more HylD and UGL in SDSE.

### Growth in Chemically Defined Minimal Medium

**Figure 3:**
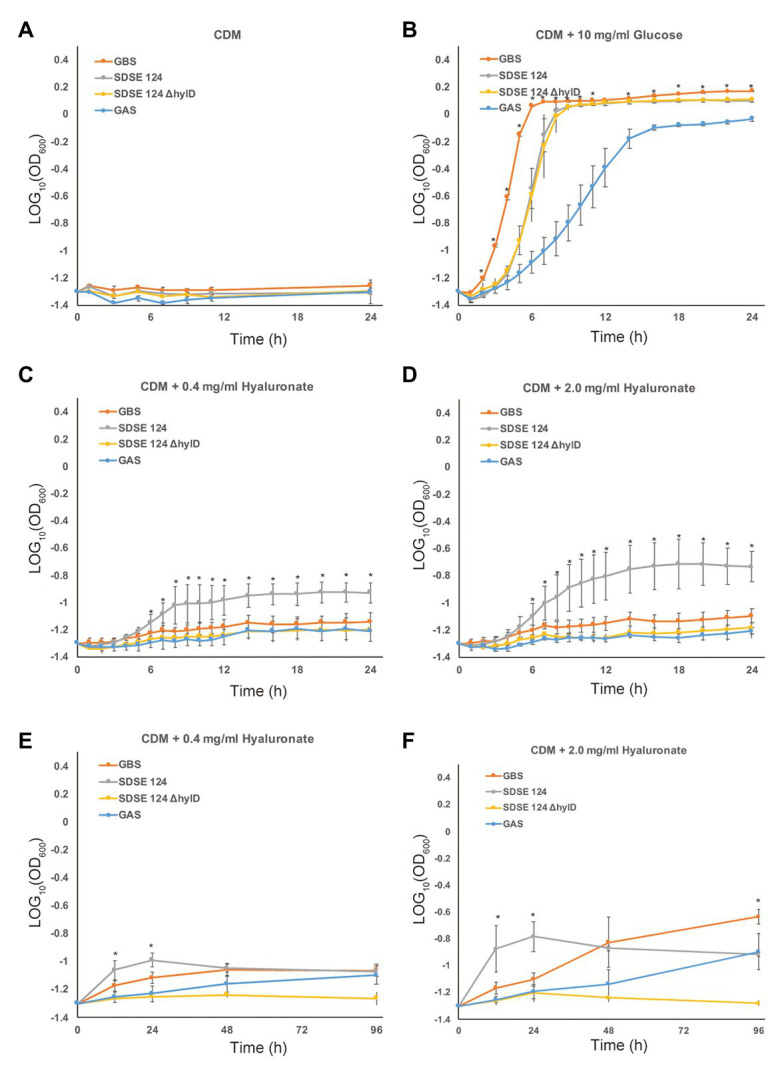
Growth curves in minimal medium. Bacterial growth was assessed during a 24 h of incubation in chemically defined medium (CDM) containing **(A)** no carbohydrate, **(B)** 10 mg/ml glucose, **(C)** 0.4 mg/ml HA, or **(D)** 2.0 mg/ml HA. Bacterial growth was assessed during a 96 h incubation in CDM containing **(E)** 0.4 mg/ml HA or **(F)** 2.0 mg/ml HA. Bacterial strains GAS-476, GBS-COH1, SDSE-124, and SDSE-124Δ*hylD* were cultivated in BHI medium overnight, collected by centrifugation, and resuspended in each medium as indicated. The average and standard deviation at each point were calculated from three separate experiments performed in triplicate. Analysis of variance and Bonferroni *post hoc* test were conducted to assess overall difference in OD_600_ at each time point; ^*^ *p* < 0.01.

Next, we analyzed the growth rates of GAS, GBS, and SDSE strains in chemically defined minimal media (CDM). None of the bacteria grew in the CDM in the absence of added carbohydrate (Figure 3A). In the presence of 10 mg/ml glucose, GBS-COH1 entered its exponential phase after 4–6 h of incubation and expanded at a rate that exceeded that of SDSE-124 wild-type and SDSE-124 Δ*hylD* mutant bacteria (Figure 3B). SDSE-124 wild-type, but not Δ*hylD* mutant, entered the exponential phase after 6–10 h of incubation and expanded at a rate that exceeded those of GBS-COH1 and GAS-476 in the presence of low (0.4 mg/ml) or high (2.0 mg/ml) concentrations of HA (Figures 3C,D). After a prolonged incubation (96 h), the OD_600_ of GBS-COH1 and GAS-476 at the plateau phase were equal to or higher than that determined for SDSE-124 (Figures 3E,F). These results indicate that SDSE can grow and divide more rapidly using HA as a carbon source than the other β-hemolytic streptococci evaluated here. OD_600_ of SDSE decreased after 48–96 h of incubation, suggesting that SDSE grows rapidly but reaches a plateau sooner (24 h).

### Enzymatic Activity

**Figure 4:**
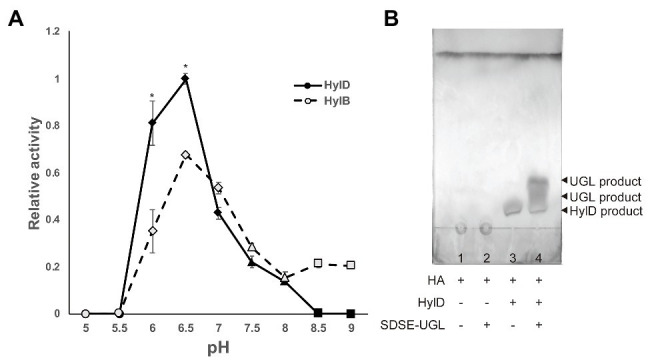
Enzymatic activities of Hyl and degradation profiles for UGL. **(A)** pH-dependent activities of Hyl proteins from GBS (HylB) and SDSE (HylD). Ten microliters of purified HylB and HylD (0.05 mg/ml in 5 mM HEPES-NaOH, pH 7.0) was added to solutions of 500 μl of 1 mg/ml HA in 0.2 M citrate (circle), MOPS-NaOH (rhomb), HEPES-NaOH (triangle), or glycine-NaOH (square). Relative activity was calculated as the increase in OD_235_ over time. *P* values were calculated using Student’s *t*-test; ^*^ *p* < 0.01. **(B)** Product analysis using thin layer chromatography. The HA solution (1 mg/ml) was incubated with PBS (1), 5 μg/ml SDSE-UGL (2), 5 μg/ml HylD (3), or both 5 μg/ml SDSE-UGL and 5 μg/ml HylD (4) at pH 6.0 (0.1 M citrate). The products were then spotted on a plate, separated with thin layer chromatography using a solvent system of 1-butanol/acetic acid/water (4:1:1, v/v), and visualized by spraying them with 5% sodium phosphomolybdate n-hydrate.

The enzymatic activity of HylD has not yet been characterized. To measure the enzymatic activities of the Hyl proteins *in vitro*, we overexpressed HylB and HylD in *E. coli* and purified them from bacterial lysates. As shown in Figure 4A, the optima for both HylB and HylD was pH 6.5; interestingly, the enzymatic activity of HylD at this pH was significantly higher than that of HylB, and the activity of HylD was twice that of HylB at pH 6.0. The enzymatic activity of HylB was slightly higher than that of HylD at pH 7.5, which approximates the physiologic pH of human blood (pH 7.4). HylD was inactive at alkaline pH (pH 8.5 and 9.0), whereas HylB retained some of its activity. A surface skin pH of 6.0 has been reported for elderly individuals and patients diagnosed with diabetes mellitus. These data suggest that HylD may show significant HA-degrading activity on skin tissue at this pH. As shown in Figure 4B, UGL acted on Hyl-produced disaccharides but not on full-length HA.

### Expression of hylD and UGL in Mice

**Figure 5:**
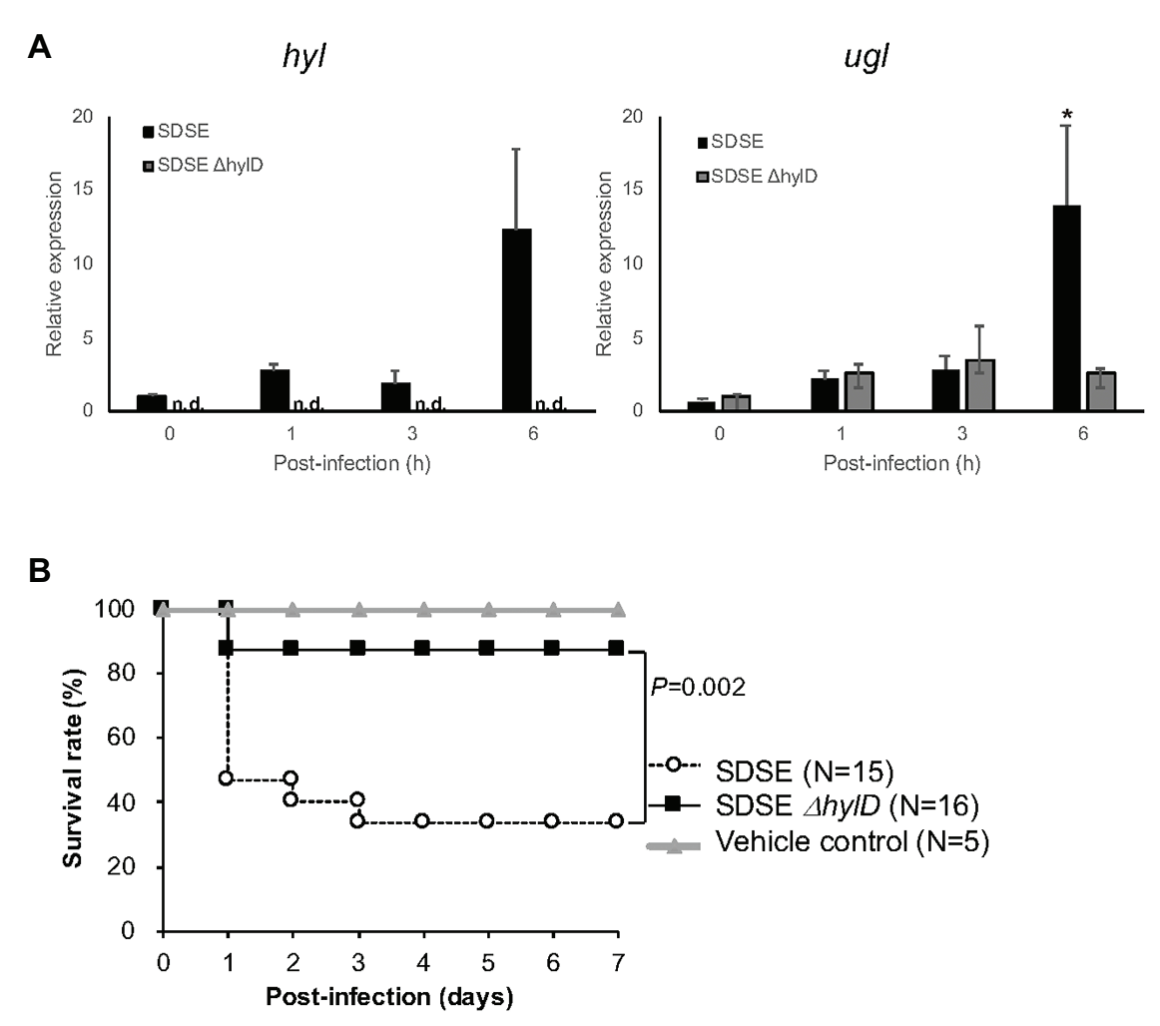
Impact of *hylD* gene deletion on SDSE infection in mice. **(A)** The relative expression of *hylD* and *ugl* after intraperitoneal injection and recovery. SDSE-124 and SDSE-124Δ*hylD* were injected intraperitoneally and collected at the indicated times; ^*^ *p* < 0.01 according to Student’s *t*-test. Relative expression levels were calculated as 1 using the averages of *hyl* and *ugl* expression in SDSE-124 at 0 h. **(B)** Lethal responses to SDSE-124 and SDSE-124Δ*hylD*. SDSE-124 and SDSE-124Δ*hylD* were injected intraperitoneally at 2.0 × 10^6^ CFU/mouse, and survival was monitored for 7 days. The value of *p* was calculated using the log-rank test. BHI medium was injected for vehicle control.

As shown in Figure 2C, HA induced the expression of both *hyl* and *ugl* genes in SDSE in experiments conducted *in vitro*. We proceeded to analyze the expression of these two genes in bacteria used to infect mice. SDSE-124 was injected into the intraperitoneal cavity of mice and collected at the hours post-inoculation as indicated. We observed significant upregulation of *hylD* in SDSE-124 recovered from mice after 6 h (Figure 5A, left panel). As anticipated, no expression was detected in SDSE-124Δ*hylD* recovered at this same time point. The gene encoding *ugl* was also upregulated in SDSE-124 that were recovered at 6 h; no upregulation was detected in recovered SDSE-124*ΔhylD* cells (Figure 5A, right panel); these results suggested that SDSE-124 coordinately regulates these genes so that HA is completely eliminated. The fact that we observed no *ugl* upregulation in the SDSE-124Δ*hylD* mutant indicates that either (a) disaccharides produced by HylD triggers *ugl* expression or (b) *ugl* expression is triggered by HylD itself.

### Lethal Infection With SDSE

Infection with SDSE-124 and SDSE-124Δ*hylD* was compared in response to intraperitoneal injection of equivalent numbers of bacteria into C57BL6/J mice. We found that 53% of the mice inoculated with SDSE-124 (eight of 15 mice) died within 1 day post-infection; 67% mortality was observed at 7 days post-inoculation (Figure 5B). By contrast, only 13% of the SDSE-124Δ*hylD*-inoculated mice (two of 16 mice) died overall. Survival was significantly higher in response to infection with the SDSE-124Δ*hylD* strain; these results suggest that HylD is a critical element promoting the pathogenicity of SDSE.

### Skin Wound Infection With SDSE

**Figure 6:**
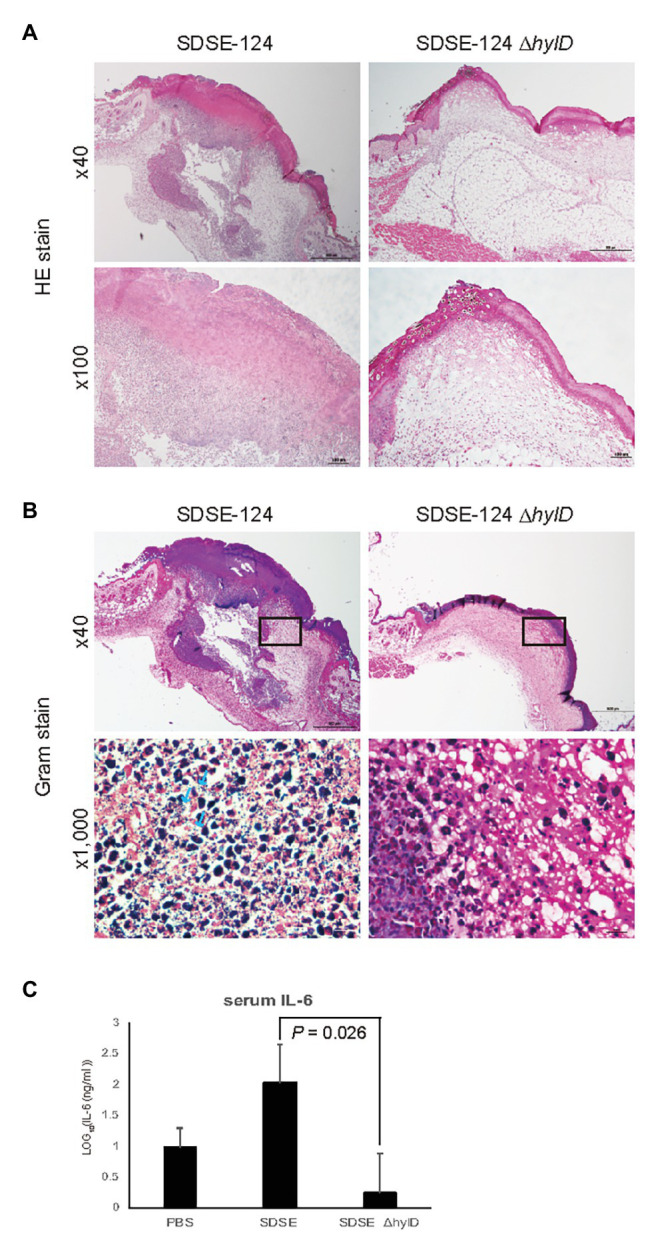
Wound infection. **(A)** H&E staining and **(B)** Gram staining of tissue sections taken from the wound areas. An arrow indicates the bacteria cells. **(C)** IL-6 concentrations of SDSE-infected mice. Data represent the mean ± SD of log-transformation of IL-6 concentrations. Mice were infected with SDSE strains (1.4 × 10^6^ CFU/mouse) *via* the wound areas. On day 2 after infection, the mice were euthanized with Sevofrane, blood was immediately collected for IL-6 tests, and samples of the wound areas were taken for H&E and Gram stains. The value of *p* was calculated using Student’s *t*-test.

We determined whether HylD is involved in skin infection considering that SDSE is omnipresent on human skin. SDSE cannot invade normal mouse skin because it lacks the *speB* gene that encodes for the cysteine protease SpeB, which is critically important for mouse skin invasion (Lukomski et al., 1999; Sumitomo et al., 2018). In this study, we tested a skin wound infection model with SDSE. Wounds were made with a biopsy punch, covered with dressing to maintain a moist environment for 24 h, and infected with SDSE-124 or SDSE-124Δ*hylD* for 2 days. Intensive infiltration of neutrophils was observed around the wound areas infected with SDSE-124 (Figure 6A), while fewer neutrophils were infiltrated around the areas with SDSE-124Δ*hylD*. Gram staining revealed many SDSE-124 cells, but not SDSE-124Δ*hylD* cells, at the wound areas (Figure 6B). Consistent with neutrophil filtration, serum IL-6 levels were significantly higher in SDSE-124-infected mice than in SDSE-124Δ*hylD*-infected mice (Figure 6C). These results suggest that HylD is involved in the inflammation of wounded skin.

## Discussion

Although SDSE is among the normal human flora, this bacterial species has also been associated with a significant diversity of diseases. Specifically, SDSE has been associated with the pathogenesis of pharyngitis, acute post-streptococcal glomerulonephritis, and acute rheumatic fever (Haidan et al., 2000; Bassett et al., 2009). While GAS and GBS possess a cluster of three genes (*hasA*, *hasB*, and *hasC*) related to HA synthesis, SDSE lacks *hasA* and *hasB* genes, resulting in absence of HA capsules. Although the clinical features of SDSE-associated diseases closely resemble GAS infections, the patient characteristics differ substantially (Watanabe et al., 2016). Notably, SDSE is a major cause of invasive β-streptococcal infections among the elderly in Japan (Takahashi et al., 2010). Most patients who present with SDSE bacteremia have other co-morbidities such as malignant tumors and diabetes mellitus (Broyles et al., 2009; Rantala, 2014). We previously explored physiologic responses to these bacteria and found that SDSE is particularly lethal in diabetic mice (Ogura et al., 2018). In this study, we focused on the relationship between HA degradation and pathogenic disease associated with SDSE. HA concentrations vary significantly among biological fluids and tissues (Cowman et al., 2015). The human skin contains approximately 0.4 mg/g HA, primarily found in the dermis (Tammi et al., 1994). HA concentration within the human knee joint is higher (2–3 mg/ml) than in any other fluid or tissue in the human body (Balazs et al., 1967; Dahl et al., 1985). SDSE is often isolated from the skins and joint fluids (Wajima et al., 2016; Matsue et al., 2020).

*Streptococcus dysgalactiae* subsp. *equisimilis* hyaluronate lyase (HylD) is homologous to GBS (HylB) at a 44% amino acid sequence identity. Because of this homology, the virulence factor database recognizes the SDSE *hylD* gene as analogous to the GBS *hylB* gene (Chen et al., 2004). Homology modeling indicated that surface charge characteristic of the modeled HylD protein resembles that of HylB despite significant differences in computed pIs (5.5 and 8.8 for HylD and HylB, respectively, Figure 1C). However, the two enzymes differ with respect to enzymatic activity (Figure 4A). In human skin, HA is localized not only in cellular matrix whose pH is neutral but also surface skin such as stratum corneum whose pH is acidic (Sakai et al., 2000). Neither enzyme can digest HA at pH 5.0 nor at pH 5.5, which is the typical pH for the surface skin of younger individuals. HylD was twice as active as HylB at pH 6.0, which is the typical pH for an aged surface skin. This increase in pH may relate to reduced expression of the sodium-hydrogen exchanger (NHE1) and filaggrin associated with reductions in acidifying free fatty acids, sebum, and sweat (Choi et al., 2007; Levin and Maibach, 2008). Surface skin pH is also increased among those diagnosed with diabetes mellitus (Behm et al., 2012). Our data suggest that HylD could degrade HA on the skins of elderly individuals and those who diagnosed with diabetes mellitus are highly susceptible to SDSE infection.

As shown in Supplementary Figure S3, *hylB* and *hylD* are designated in the genomes differently among GBS and SDSE. We determined that SDSE can degrade HA more effectively than do equal amounts of GBS and GAS; this observation relates to augmented production and secretion of HylD and UGL (Figure 2). We also found that SDSE grew rapidly in chemically defined minimal media containing HA as the sole carbohydrate source (Figure 3). Moreover, expression of *hylD* gene was significantly upregulated in SDSE injected intraperitoneally in mice (Figure 5A). HylD degrades HA *via* a β-elimination reaction to unsaturated disaccharides, which are then hydrolyzed by UGL. As shown in Figure 5A, SDSE-124Δ*hylD* did not upregulate the *ugl* gene, suggesting the accumulation of unsaturated disaccharides may trigger its expression; a further understanding of this mechanism might be pursued in future studies. Of note, Kolar et al. reported that unsaturated disaccharides generated in GBS block inflammatory responses and permit the bacterial cells to escape immune surveillance (Kolar et al., 2015). Following this line of thinking, high concentrations of UGL may lead to a decrease in the concentration of accumulated disaccharides and thus promote inflammatory responses; this will represent a significant disadvantage from the perspective of bacterial evasion.

It remains unclear as to whether disaccharides generated by the actions of HylD can limit inflammation and promote SDSE-124 immune evasion.

We found that deletion of *hylD* gene resulted in a significant decrease in mortality among infected C57BL6/J mice (Figure 5B). Moreover, in open wounds, SDSE wild-type but not Δ*hylD* mutant resided on day 2 post-infection, resulting in infiltration of neutrophils and increase of serum IL-6 level (Figure 6). These results suggest that HylD is involved in the pathogenicity of SDSE through nutrient degradation on host skins. By contrast, deletion of the *hylB* gene in GBS resulted in amplified lethality that was eliminated in infection studies conducted in *Tlr2* gene knockout mice (Kolar et al., 2015). Of note, GBS but not SDSE secrete hyaluronate capsules that promote immune evasion; deletion of the *hylB* gene in GBS may promote formation of a more substantial capsule and thereby promote lethal infection.

It remains unclear how SDSE typically found on the skin invades and enters systemic circulation to generate life-threatening bacteremia. SDSE does not produce cysteine protease SpeB, which is a critical element that promotes GAS invasion (Lukomski et al., 1999; Sumitomo et al., 2018). It is possible that SDSE does not transit through normal skin but can invade *via* sites of injury sites. Frenkel (2014) reported that HA is an integral component at the site of skin injury and promotes wound healing, indicating that SDSE can target HA accumulated at the healing sites. Further analysis will be required to elucidate the mechanism of SDSE invasion.

In conclusion, we characterized the expression of genes encoding for HylD and UGL in bacterial strain SDSE-124. These two enzymes were upregulated in response to HA. This investigation suggested that HylD and UGL play important roles in nutrient acquisition from hosts, followed by the bacterial pathogenicity damaging host tissues. This is the first report that has identified and characterized a specific virulence factor from SDSE, to the best of our knowledge.

## Data Availability Statement

The original contributions presented in the study are included in the article/Supplementary Material, further inquiries can be directed to the corresponding author.

## Ethics Statement

The animal study was reviewed and approved by the Committee for Genetic Recombination Experiments (Kanazawa University).

## Author Contributions

VAN performed the experiments. KO conceived this project, directed the study, and conducted enzymatic analysis. VAN and KO wrote the manuscript. MM contributed to mice experiments and improved the quality of the manuscript. NT contributed to study design for revision of the manuscript. KM contributed to design of wound infection experiments. YN contributed to sample preparation for histological analysis. TLH, YI, NS, and TW contributed to histological analysis of skin tissues. WH critically revised the manuscript and contributed to hyaluronate degradation assay and TLC analysis. SO contributed to ethical approval of the animal experiments and progress of the study. HI supervised VAN and directed the study. All authors contributed to the article and approved the submitted version.

### Conflict of Interest

The authors declare that the research was conducted in the absence of any commercial or financial relationships that could be construed as a potential conflict of interest.

## Footnotes

^1^http://tree.bio.ed.ac.uk/software/figtree/
