# Supplementary material for: Novel Hyaluronate Lyase Involved in Pathogenicity of Streptococcus dysgalactiae subsp. equisimilis
Source: Front Microbiol. 2020 Sep 24;11:552418. doi: 10.3389/fmicb.2020.552418 (PMC7541959; doi:10.3389/fmicb.2020.552418)
Supplement: Supplementary file 1 [file Data_Sheet_1.pdf]

*Supplementary Material*

# Novel Hyaluronate Lyase Involved in Pathogenicity of *Streptococcus dysgalactiae* subsp. *equisimilis*

Van An Nguyen, Kohei Ogura, Miki Matsue, Norihiko Takemoto, Kanae Mukai, Yukari Nakajima, Thuy Linh Hoang, Yasunori Iwata, Norihiko SakaiTakashi, Wada, Wataru Hashimoto, Shigefumi Okamoto, Hiroshi Ichimura

*** Correspondence:** Kohei Ogura: ogura@staff.kanazawa-u.ac.jp

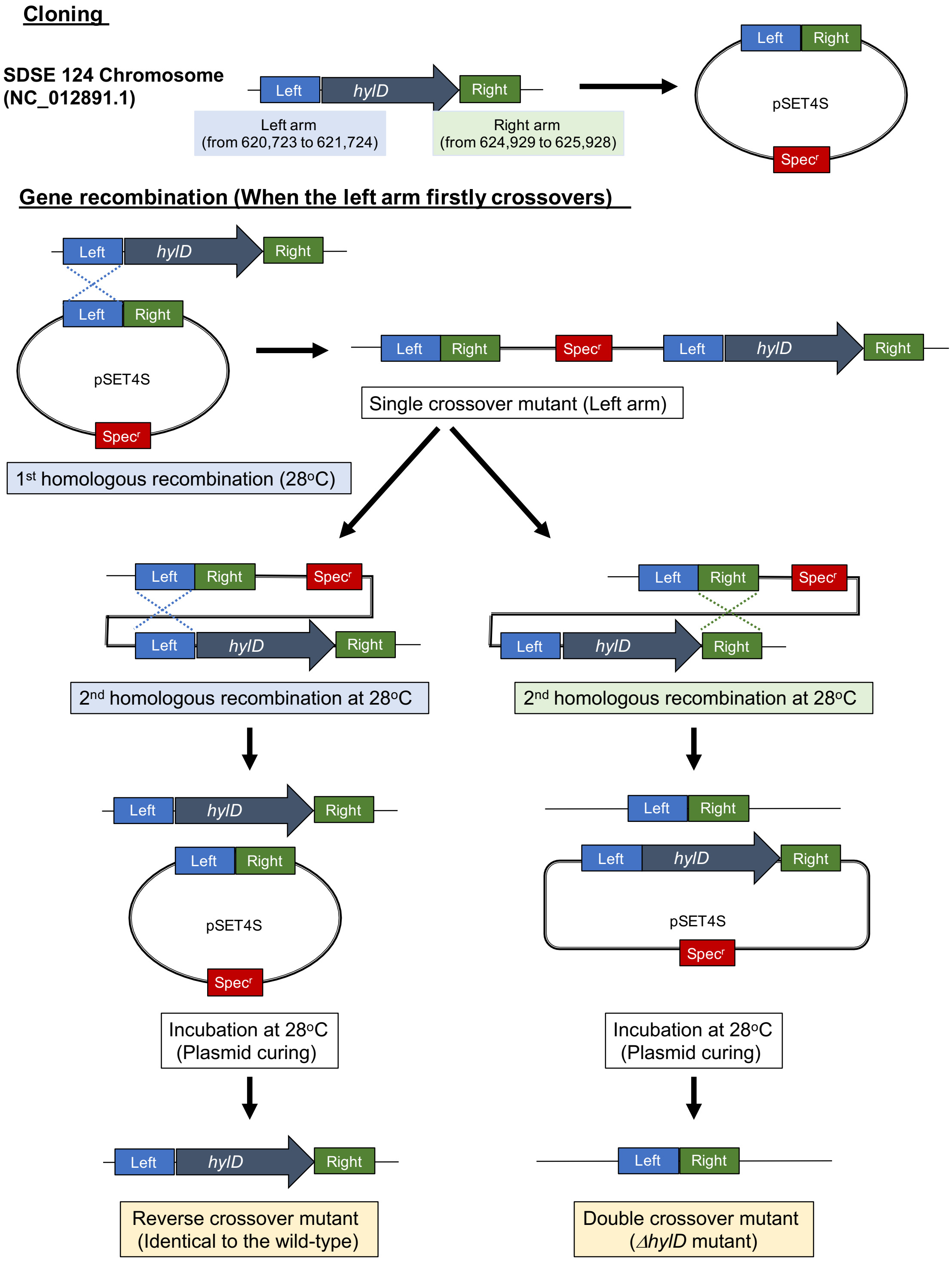


**Figure S1. Diagram of procedure for prepare Δ*hylD* mutant strain.** While this picture only shows a case when the first crossover mutant is obtained by homologous recombination of left arm (1 kb upstream sequences of the *hylD* gene in the SDSE chromosome), recombination of right arm (1 kb downstream sequences) also occurred. After transformation of the SDSE-124 by electroporation (1.75 kV pulse), All of the procedures were conducted at room temperature or 28ºC.

```
HylB   MEIKKKHRIMLYSALILGTILVNNSYQAKAEELTKTTSTSQIRDTQTNNIEVLQTESTTV
HylD   -MKHKKYYPYICSSILLACLLWSPTVLADEDSSGTTALPMPQEIEPPQQITDLSHQVTSQ
          :**:   : *:::*. :* . :   *. :.  .*: .    .    .::*  *. : *:

HylB   KETSTTTTQQDLSNPTASTATATATHSTMKQVVDNQTQNKELVKNGDFNQTNPVSGSWSH
HylD   NDVAVTTTEKEGDSDLESQVTLTGENLLKNPQFDQTSPASTTSTDKDK--------GWSK
       ::.:.***::: ..    * .* *. :    :  .*: :  .    .: *          .**:

HylB   TSAREWSAWIDKENTADKSPIIQRTEQGQVSLSSDKGFRGAVTQKVNIDPTKKYEVKFDI
HylD   EAAEGWQVYKDSKQTVGSPQIDASEQQLTMTNEAGKKLRGCVHQTVVINPEKQYLVSFDI
        :*. *..: *.::*.... *     :*  :: .:.* :**.* *.* *:* *:* *.***

HylB   ETSNKAGQAFLRIMEKKDNN-----TRLWLSEMTSGTTNKHTLTKIYNPKLNVSEVTLEL
HylD   ETKDKIGQAFVRVIEEIKENNTLKEQRLWLSPMATGTMKKH-QEKLYVPKLKVNQIKLEL
       **.:* ****:*::*: .:*       ***** *::** :**   *:* ***:*.::.***

HylB   YYEKGTGSATFDNISMKAKGPKDSEHPQPVTTQIEESVNTALNKNYVFNKADYQYTLTN-
HylD   FYETGQGQVIFDNISLREAGDKPSGAIKEVTHSLEERIALSLSKKHLLAIPDYIYQVAAG
       :**.* *.. *****::  * * *    : ** .:** :  :*.*::::  .** * ::

HylB   -PSLGKIVGGILYPNATGSTTVKISDKSGKIIKEVPLSVTASTEDKFTKLLDKWNDVTIG
HylD   ANQIVRIDNGTVEPLQEGHTLLEVFTKEGQKIADLPLEVTGKDDSEMTALISQWRQMILG
          .: :* .* : *    * * :::  *.*: * ::** :.::* *:.:*.:: :*

HylB   NHVYDTNDSNMQKINQKLDETNAKNIKTIKLDSNHTFLWKDLDNLNNSAQLTATYRRLED
HylD   ADSYSVSNPAMQALNQKLDDSVTKNLNSLVNNQKESYLWEDLKDFGKSSHMTATYRRLEE
         . *...:. ** :*****:: :**::::  :.:.::**:**.::.:*:::********:

HylB   LAKQITNPHSTIYKNEKAIRTVKESLAWLHQNFYNVNKDIEGSANWWDFEIGVPRSITAT
HylD   MAKQVNSPASKYYQDTALIRLIKDKLAWLHLNYYNPQKDIEGNANWWDYEIGTPRAIVNT
       :***:..* *. *::   ** :*:.***** *:** :*****.*****:***.**:*. *

HylB   LALMNNYFTDAEIKTYTDPIEHFVPDAGYFRKTLDNPFKALGGNLVDMGRVKIIEGLLRK
HylD   LTLLYPYFTQDEIKAQTKSISHFVPDPKQFRSTLVNPFKAIGGNLVDMGRVKIIEALLTQ
       *:*:  ***: ***: *..*.*****.  **.** *****:**************.** :

HylB   DNTIIEKTSHSLKNLFTT---ATKAEGFYADGSYIDHTNVAYTGAYGNVLIDGLTQLLPI
HylD   DDVKLKESIEALDTLFEFQRDGSKGEGFYKDGSYIDHTNVAYTGAYGNVLIDGLSQLIPL
       *:. :::: .:*..**      .:*.**** ************************:**:*:

HylB   IQETDYKISNQELDMVYKWINQSFLPLIVKGELMDMSRGRSISREAASSHAAAVEVLRGF
HylD   IQASPMALNAQKLEVIEHWIEKSFFPLLIHGELIDMSRGRSISRENASSRMAALEALRGI
       ** :   :. *:*::: :**::**:**::***:*********** ***: **:*.***:

HylB   LRLANMSNEERNLDLKSTIKTIITSNKFYNVFNNLKSYSDIANMNKMLNDSTVATKPLKS
HylD   LRLSQVLPEANKNRIQGQLKSILAFHDKERMLSSLSSYYDINLFEQVLANEVIKLMPMST
       ***:::  * .:  ::. :*:*:: :.  .::..*.** **  ::::* :..:   *:.:

HylB   NLSTFNSMDRLAYYNAEKDFGFALSLHSKRTLNYEGMNDENTRDWYTGDGMFYLYNSDQS
HylD   NLSVFQNMDKLAYYHTDKDFGFALSMHSNRTLNFEAMNNENTRGWYTGDGMFYLYNHDLT
       ***.*:.**:****:::********:**:****:*.**:****.************ * :

HylB   HYSNHFWPTVNPYKMAGTTEKDAKREDTTKEFMSKHSKD----AKEKTGQVTGTSDFVGS
HylD   HYSDKYWPTVNPLKMPGTTEAEGKREDVTDDYLKKLTIDYKEKAKEEAGMSTLPSSFVGA
       ***::******* **.**** :.****.*.::::* : *    ***::*  * .*.***:

HylB   VKLNDHFALAAMDFTNWDRTLTAQKGWVILNDKIVFLGSNIKNTNGIGNVSTTIDQRKDD
HylD   IKGDDKTALAAMDFQNWDRTVSAKKAWGIFDDRIVFLGAGIQSTKDQ-AVSTTIDQRKDN
       :* :*: ******* *****::*:*.* *::*:*****:.*:.*:.    **********:

HylB   SKTPYTTYVNGKTIDLKQASSQQFTDTKSVFLESKEPGRNIGYIFFKNSTIDIERKEQTG
HylD   PENPYRLLVNGQEVSLTNDT-LEKDHVTSVLLLSQDGKNNIGYLFDKPTTLVFSRQEQSG
       .:.**    ***: :.*.: :  :   ...**:* *::  .****:* * :*: :.*:**:*

HylB   TWNSINRTSKNTSIVSNPFITISQKHDNKGDSYGYMMVPNIDRTSFDKLANSKEVELLEN
HylD   RWSDINKGSTNKELVTQTFITISQRHQQANDTYAYTLLPNVSQEDFDKARTESSIEVVRN
        *..**: *.*..:*::.******:*::  .*:*.* ::**:.: .***  ....:*::.*

HylB   SSKQQVIYDKNSQTWAVIKHDNQESLINNQFKMNKAGLYLVQKVGNDYQNVYYQPQTMTK
HylD   DSDLQILHDHKQDLWTVVNYHDGPQRINDQLTLEKAGLYLYQKVGNVFKLLSKDLLPSD-
       .*. *:::*::.: *:*::::  . **:*:.::******* ***** :: :  :   .

HylB   TDQLAI
HylD   ------
```

**Figure S2.** Alignments of HylB (from GBS strain COH1) and HylD (from SDSE strain 124) proteins using Clustal W. Catalytic residues were colored by yellow. The loop region ($_{804}$KKLTID$_{809}$) was indicated by blue.

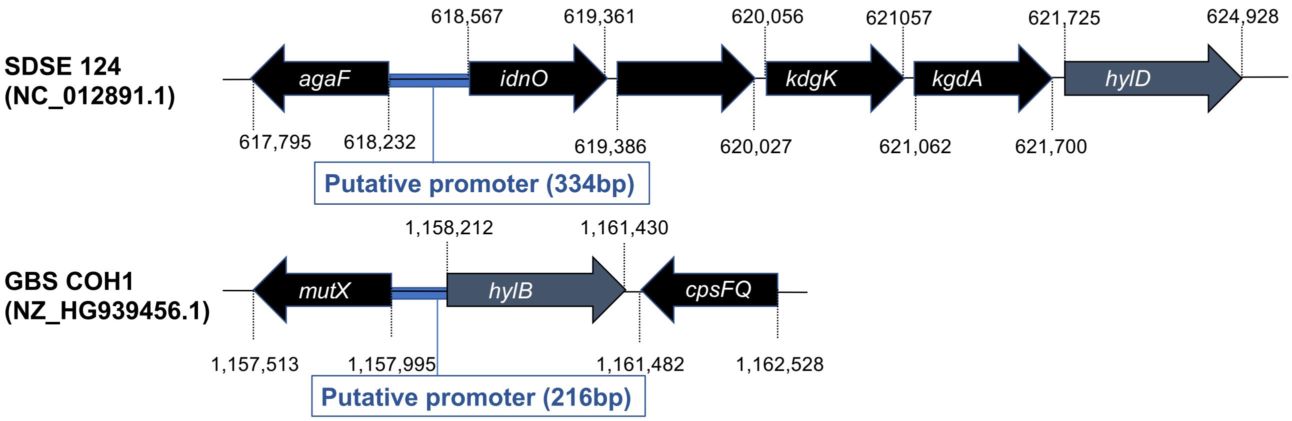


**Figure S3. Genetic arrangements of *hylD* and *hylB* genes.** Putative promoter regions were indicated in blue.
